# A reassessment and comparison of the Landolt C and tumbling E charts in managing amblyopia

**DOI:** 10.1038/s41598-021-97875-3

**Published:** 2021-09-14

**Authors:** Yu-Hung Lai, Horng-Jiun Wu, Shun-Jen Chang

**Affiliations:** 1 Department of Ophthalmology, Kaohsiung Medical University Hospital, Kaohsiung Medical University, 100 Zih-You 1st Road, Kaohsiung, 80708 Taiwan; 2 Department of Ophthalmology, School of Medicine, College of Medicine, Kaohsiung Medical University, Kaohsiung, 80708 Taiwan; 3 Graduate Institute of Medicine, College of Medicine, Kaohsiung Medical University, Kaohsiung, 80708 Taiwan; 4 Department of Kinesiology, Health and Leisure Studies, National University of Kaohsiung, Kaohsiung, 81148 Taiwan

**Keywords:** Health policy, Vision disorders, Paediatric research

## Abstract

Current criteria for amblyopia do not account for difference in visual acuity charts. This prospective observational study analyzed 100 children younger than 10 years treated at a tertiary referral center. Visual acuity was separately tested in each eye using Landolt C and tumbling E charts in a random order. For each chart, receiver operating characteristic curve analysis was performed to determine the best cutoff for visual acuity score. Main outcome measures included the difference in visual acuity scores between the two charts, the feasibility of repeated testing of visual acuity in each eye, and amblyopia cutoff values for each chart. Mean logMAR visual acuity scores obtained by tumbling E chart were significantly better than those obtained by Landolt C chart. For amblyopia, the best cutoff values were <  + 0.14 (20/27 Snellen equivalent) for tumbling E chart and <  + 0.24 (20/35 Snellen equivalent) for Landolt C chart. For children under 10 years old, visual acuity scores for tumbling E chart were significantly better than those for Landolt C chart. We suggest that amblyopia management in children should account for age and the type of visual acuity chart used.

## Introduction

Reduced visual acuity is a diagnostic criterion for amblyopia. The diagnostic criteria for amblyopia in the Amblyopia Preferred Practice Pattern (PPP) implemented by the American Academy of Ophthalmology are presence of at least one amblyopia risk factor and visual acuity worse than 20/50 for children aged 3 to ≤ 4 years, worse than 20/40 for those aged 4 to ≤ 5 years, and worse than 20/30 for those aged > 5 years^1^. Similar guidelines are observed in Taiwan^2^. In children, however, different visual acuity charts may obtain different visual acuity scores^3–5^. Since the type of visual acuity chart is not specified in the guidelines, there is an unresolved gap whether visual acuity chart dependent criteria for amblyopia are required.

In eye clinics in Taiwan, the Landolt C and tumbling E charts are the most widely used visual acuity charts for all ages, from preschoolers to adults. Visual acuity charts such as the ETDRS chart are inapplicable in Taiwan and many other countries in which the writing system is not based on the Latin alphabet. Even in countries that use the Latin alphabet, the ETDRS chart has relatively low testability in preschool children^6^. Our previous studies in a Taiwan population indicate that the Landolt C and tumbling E charts are feasible for use in vision screening in this age group^2,5^; another recent study of a European Caucasian population similarly found that the tumbling E chart is applicable in this age group^7^.

Since development of visual acuity continues throughout early childhood, the visual acuity scores in adults should not be regarded as equivalent to that of children, or as a reference for managing amblyopia. Treacy et al. recently reported that comparing mean visual acuity scores in adults significantly differed between the tumbling E chart and the Landolt C chart^8^, however, there is no good study in the literature whether the tumbling E chart and the Landolt C charts give the same scores in children. Our previous study found that, for children in this age group, mean visual acuity scores given by the tumbling E chart were significantly better than those given by the Landolt C chart^5^. However, a noted disadvantage of the study was that each of the two visual acuity charts had been used in different populations and under different clinical conditions. Therefore, it is important to understand the conditions of use of the two charts in children.

Based on our clinical experience, we hypothesized that different visual acuity charts obtain discrepant visual acuity scores when used in children. If our hypothesis is true, different amblyopia criteria must be established for different visual acuity charts and for different age groups. Few studies have investigated this issue. Thus, the objective of this study was to determine whether the Landolt C and tumbling E charts obtain different visual acuity scores in a population of children younger than 10 years. The second objective was to use receiver operating characteristic (ROC) curve analysis to determine the best cutoff scores on the Landolt C and tumbling E charts for amblyopia diagnosis in this age group.

## Results

### Demographic characteristics

The male–female ratio was 1.08. Mean age was 65.7 months (95% confidence interval [CI]  62.6–68.8; age range 41–117 months). Mean spherical equivalent (SE) was 0.42 D (95% CI − 0.10 to + 0.93; range − 8.75 to  + 7.25 D) for the eye tested first and 0.31 D (95% CI − 0.24 to  + 0.86; range − 9.38 to  + 6.75 D) for the eye tested second. Mean astigmatism was 1.46 D (95% CI 1.23–1.69; range 0.00–4.00 D) for the eye tested first and 1.46 D (95% CI 1.23–1.69; range 0.00–4.50 D) for the eye tested second. In total, 38% of the children had amblyopia. The types of amblyopia were refractive (45%), strabismic (37%), ansiometropic (37%) and deprivation (11%). Twenty-six percent of the children had a combined mechanism of amblyopia. Of the 38 amblyopic children, 32 had been treated previously and 6 were new patients. Previous treatments included refractive correction, occlusion therapy, and/or surgery depending on the amblyopia type. Additionally, 21 had a systemic disorder or disease; 2 had attention deficit disorder; 8 had allergic rhinitis, asthma, or atopic dermatitis; 6 had developmental delay; 2 had tic disorder; 1 had dizziness; 1 had Henoch–Schlonlein purpura; and 1 had received surgery for recurrent urethrocutaneous fistula.

### Comparison of Landolt C and tumbling E charts

**Figure 1:**
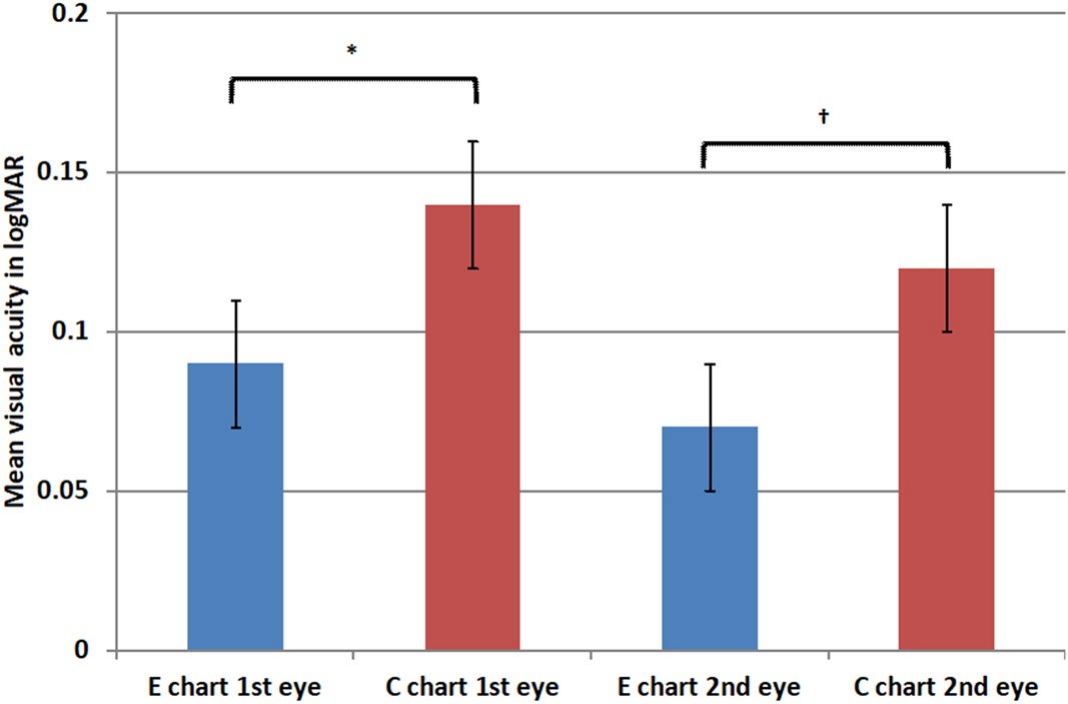
Comparison of visual acuity scores obtained by Landolt C and tumbling E charts. *For the eye tested first, the mean visual acuity score for the tumbling E chart was significantly better than that for the Landolt C chart (p < 0.001). ^†^For the eye tested second, the mean visual acuity score for the tumbling E chart was also significantly better than that for the Landolt C chart (p = 0.003).

Fifty children were tested by Landolt C chart first, and the other 50 children were tested by tumbling E chart first. In the 100 children, 20 (20%) of eyes tested first were amblyopic while 22 (22%) of eyes tested second were amblyopic. Figure 1 shows that, for eyes tested first, the tumbling E chart obtained significantly better logMAR mean visual acuity scores compared to the Landolt C chart (+ 0.09 vs. + 0.14, respectively; p < 0.001). For eyes tested second, the tumbling E chart also obtained significantly better logMAR mean visual acuity scores compared to the Landolt C chart (+ 0.07 vs. + 0.10, respectively; p = 0.003). When the comparison was limited to subjects aged < 84 months, the difference in visual acuity scores between the Landolt C chart and the tumbling E chart was still significant (p < 0.001).

### Effect of testing sequence in the same eye

In eyes tested first, scores obtained when the tumbling E chart was used first did not significantly differ from scores obtained when Landolt C chart was used first (i.e., logMAR visual acuity by tumbling E chart-logMAR visual acuity by Landolt C chart; p > 0.05; − 0.07 ± 0.12 when tumbling E chart was used first; − 0.03 ± 0.07 when Landolt C chart was used first). Similarly, in eyes tested second, scores obtained when tumbling E chart was used first (− 0.05 ± 0.14) did not significantly (p > 0.05) differ from scores obtained when Landolt C chart was used first (− 0.02 ± 0.06). That is, the sequence of charts did not significantly affect the visual acuity scores.

### Effects of repeated tests of visual acuity

**Figure 2:**
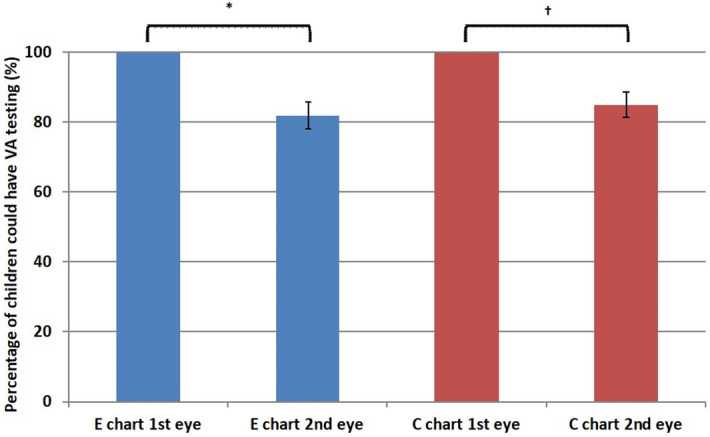
Comparison of testability between eyes tested first and second. *A significant number of children (n = 18) refused to undergo the test of the second eye by tumbling E chart (p < 0.001). ^†^A significant number of children (n = 15) refused to undergo the test of the second eye by Landolt C chart (p < 0.001).

Visual acuity scores obtained by the tumbling E chart did not significantly differ between the first eye and the second eye (p > 0.05; mean logMAR visual acuity for first eye =  + 0.09 ± 0.17; mean logMAR visual acuity for second eye =  + 0.07 ± 0.19). Although all subjects completed tests of visual acuity in the first eye, a significant number refused to undergo tests of the second eye (n = 18; p < 0.001; Fig. 2). Visual acuity scores obtained by the Landolt C chart did not significantly differ between the first eye and the second eye (p > 0.05; mean logMAR visual acuity for first eye =  + 0.14 ± 0.20; mean logMAR visual acuity for second eye =  + 0.12 ± 0.25). Similarly, while all children completed tests of visual acuity in the first eye by Landolt C chart, a significant number of children refused to undergo tests of the second eye by Landolt C chart (n = 15; p < 0.001; Fig. 2).

### Additional factors in visual acuity scores

**Figure 3:**
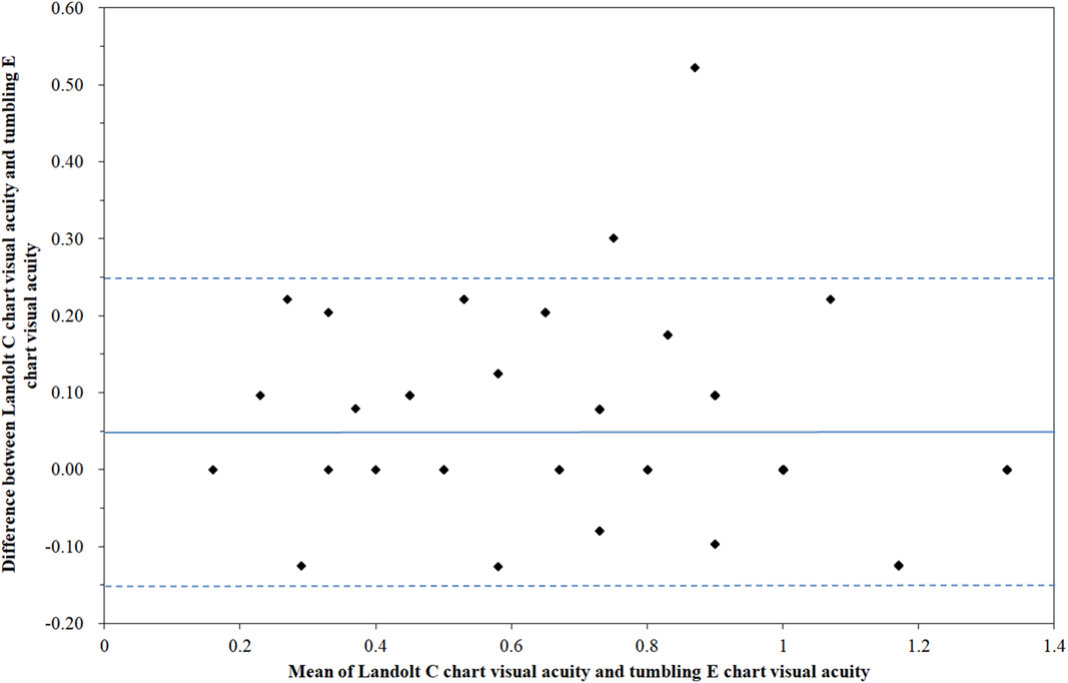
Bland–Altman plot analysis of the Landolt C and tumbling E charts. Mean of the difference (bias) was 0.05 (solid line), and the standard deviation was 0.10. The lower limit of agreement was − 0.15, and the upper limit of agreement was 0.25 (dashed lines).

The difference in visual acuity scores between the Landolt C chart and the tumbling E chart (difference in visual acuity [logMAR format] between the Landolt C chart and the tumbling E chart = [the tumbling C chart visual acuity] −[Landolt E chart visual acuity]) did not significantly differ between amblyopic eyes and non-amblyopic eyes (the difference in visual acuity scores between the two charts in amblyopic eyes was 0.06 and the difference was 0.04 in non-amblyopic eyes; p > 0.05). The testability of visual acuity in the second eye did not significantly differ by gender or by the presence of systemic disease (p > 0.05). Age had significant associations with visual acuity (in logMAR, Spearman’s rho = − 0.420 in tumbling E chart and − 0.449 in Landolt C chart; p < 0.001 and < 0.001, respectively). Additionally, scores for the tumbling E chart had significant correlations with scores for the Landolt C chart (Spearman’s rho = 0.819; p < 0.001). Figure 3 shows the Bland–Altman plot of the two charts. The mean of the difference (bias) was 0.05, and the standard deviation was 0.10. The lower limit of agreement was − 0.15, and the upper limit of agreement was 0.25. The p value in one-sample t test was less than 0.001, which indicated proportional bias between the two charts. The beta coefficient value for the linear regression was 0.159 (p = 0.004).

### Age differences in the sensitivities and specificities of various criteria

Table 1 shows the age differences in the sensitivities and specificities of various criteria. For the Landolt C chart, specificity was higher for Criterion B compared to Criterion C, while the sensitivity was the same for Criteria B and C. For the Landolt C chart, Criterion A had better specificity than Criterion B, but Criterion A had too low sensitivity (0.650). For the tumbling E chart, Criterion C had superior sensitivity and specificity (better than 0.860) while Criterion B had sensitivity of 0.800 and Criterion D had specificity of 0.787.

**Table 1:** Sensitivities and specificities of Landolt C and Tumbling E charts for varying criteria.

Criterion^a^	Visual acuity chart	Sensitivity	Specificity
A	Tumbling E^b^	–	–
	Landolt C	0.650	0.912
B	Tumbling E	0.800	0.937
	Landolt C^c^	0.950	0.787
C	Tumbling E^c^	0.900	0.863
	Landolt C	0.950	0.700
D	Tumbling E	1.000	0.787
	Landolt C^b^	–	–

^a^Criteria: (A) visual acuity (logMAR format): > 0.40 (0.4 in decimal format) at age 3, > 0.30 (0.5 in decimal format) at age 4, > 0.22 (0.6 in decimal format) at age 5, > 0.15 (0.7 in decimal format) at age 6, > 0.10 (0.8 in decimal format) at age 7, or > 0.00 (1.0 in decimal format) at age 8 and older; (B) visual acuity: > 0.30 at age 3, > 0.22 at age 4, > 0.15 at age 5, > 0.10 at age 6 or > 0.00 at age 7 and older; (C) visual acuity: > 0.22 at age 3, > 0.15 at age 4, > 0.10 at age 5, or > 0.00 at age 6 and older; (D) visual acuity: > 0.15 at age 3, > 0.10 at age 4, > 0.05 (0.9 in decimal format) at age 5, > 0.00 at age 6 and older.

^b^Sensitivity and specificity of tumbling E chart were inapplicable to criterion A. Sensitivity and specificity of Landolt C chart were inapplicable to criterion D.

^c^Chart with superior sensitivity and specificity for these criteria.

### ROC curve analysis of amblyopia criteria

For children aged < 84 months, the area under the curve was 0.966 for tumbling E chart and 0.935 for Landolt C chart. Table 2 shows that, for amblyopia diagnosis, the best cutoff values (logMAR) were <  + 0.1364 (20/27 Snellen equivalent) for the tumbling E chart and <  + 0.2385 (20/35 Snellen equivalent) for the Landolt C chart. The ROC curve analysis was inapplicable in children aged > 84 months.

**Table 2:** Best cutoff values for using tumbling E and Landolt C charts for amblyopia diagnosis in children aged < 84 months in receiver operating characteristic curve analysis.

Visual acuity chart	AUC (95% CI)	Best cutoff value in logMAR (Snellen equivalent)	p value
Tumbling E	0.966 (0.930–1.000)	+ 0.1364 (20/27)	< 0.001
Landolt C	0.935 (0.878–0.991)	+ 0.2385 (20/35)	< 0.001

*AUC* area under curve, *CI* confidence interval.

## Discussion

Our research found that, in children under 10 years old, the tumbling E and Landolt C charts obtain discrepant visual acuity scores. Specifically, in children under 10 years old, visual acuity scores measured by the tumbling E chart tended to be better than those obtained by the Landolt C chart. The difference in scores was unaffected by the sequence in which the charts were used. Interestingly, vision measurements obtained by the tumbling E chart were better than those obtained by the Landolt C chart, which was consistent with the previous findings of Treacy et al.^8^ in an adult population in the United Kingdom and with the findings of Chaikitmongkol et al. in an adult population in Thailand^9^. Reduced visual acuity is one of the criteria used for diagnosis and management of amblyopia. The 0.05 logMAR difference was significant in Bland–Altman analysis and was consistent with the differences reported in the literature for adult age groups. The significant logMAR difference yields different visual acuity cutoffs for different visual acuity charts used for diagnosing and managing amblyopia.

For the Landolt C chart, the criteria with the best age-dependent sensitivity and specificity for amblyopia diagnosis were Criterion B (visual acuity in logMAR format > 0.30 at age 3; > 0.22 at age 4; > 0.15 at age 5; > 0.10 at age 6; or > 0.0 at age 7 and older). For the tumbling E chart, the criteria that had the best age-dependent sensitivity and specificity for amblyopia diagnosis were Criterion C (visual acuity in logMAR format > 0.22 at age 3; > 0.15 at age 4; > 0.10 at age 5; or > 0.00 at age 6 and older). Neither the PPP guidelines of the American Academy of Ophthalmology in the United States nor the vision screening and correction guidelines for children established by the Health Promotion Administration, Ministry of Health and Welfare in Taiwan specify different criteria for different visual charts used for amblyopia diagnosis. Although Criterion A applied in the current study appear similar to the PPP criteria of the American Academy of Ophthalmology, the results of our study are not consistent with the amblyopia diagnostic criteria recommended by the PPP. Possible reasons for the difference include the different visual acuity charts used in different countries as well as language and cultural differences.

According to our ROC curve analysis results, the two charts differed in the best cutoff for amblyopia in patients younger than 7 years. In this age range, the cutoff for the tumbling E chart is higher than that for the Landolt C chart; that is, the tumbling E chart tends to obtain better scores. Since both charts are widely used in Taiwan, amblyopia diagnosis and management in children should consider both age and the type of visual acuity chart. Additional studies are needed to compare cutoffs for other types of visual acuity charts in this age group.

An additional finding of our study is that, even when different charts (i.e., C and E charts) are used to test visual acuity, repeated testing may reduce the testability rate in this age group. Hence, performing multiple visual acuity tests in a single day is not recommended. Given the limited attention span and tolerance of patients at this age, results for a single test of visual acuity should be interpreted cautiously. Our experience is that testability can be increased by verbal encouragement or by token rewards (e.g., stickers). In our practice, it’s not uncommon that the patients were not emotionally and/or psychologically well-prepared for the visual acuity testing. Additionally, in patients with borderline refractive error (e.g., astigmatism 1.50D), visual acuity may be only one line worse than the criterion for amblyopia. For those patients, an amblyopia diagnosis will not always be made at the same visit. Young patients and their parents often exhibit psychological resistance to the prospect of wearing glasses. Therefore, delaying the amblyopia diagnosis and spectacle prescription until further confirmation by another visual acuity test in a follow-up visit of 2 months may provide a sufficient time interval for emotional and psychological acceptance of a spectacle prescription by young patients and their parents. Notably, history of attention deficit disorder revealed no association with visual acuity score, but further studies in a larger population are needed to confirm this finding.

A strength of this study is that all subjects were recruited from an ophthalmology clinic, instead of from the general population, which increases the relevance of the findings with regard to amblyopia management. A limitation of this study is its relatively small sample size, which precluded the use of more detailed age-dependent criteria in ROC curve analysis and limits the generalizability of the findings to healthy populations.

In conclusion, for children under 10 years old, visual acuity scores measured by the tumbling E chart tended to be higher than those obtained by the Landolt C chart. Amblyopia management in children should consider their age and the type of visual acuity chart used to diagnose amblyopia. Additional studies are needed to compare amblyopia diagnostic criteria in other visual acuity charts in this age group.

## Methods

### Study subjects

This prospective observational study was performed in line with the principles of the Declaration of Helsinki. Approval was granted by the Institutional Review Board, Kaohsiung Medical University Chung-Ho Memorial Hospital (Number: KMUHIRB-20130081). Informed consent was obtained from all individual participants and/or their legal guardians. We recruited 100 children younger than 10 years during December, 2017, to November, 2018. All children were recruited during visits to the ophthalmology clinic at our hospital, and all had been referred for treatment of poor visual acuity and/or amblyopia after a vision screening performed by a preschool clinic or by a local pediatrician or ophthalmologist. Some patients had previously received occlusion therapy for amblyopia or refractive correction prescriptions at our hospital. Data collection included age, gender, refractive errors, visual acuity, ocular alignment and motility, slit lamp examination results, fundus examination results, and systemic diseases.

### Visual acuity testing

A single pediatric ophthalmologist (YHL) performed all visual acuity tests in all participants. The Landolt C and tumbling E charts in Smart System (M & S Technology, Inc, IL, USA) were used at a distance of 4 m in accordance with the manufacturer recommendations. Each increase in visual acuity score from 20/125 to 20/15 (excluding 20/70) approximated a logarithm of the minimum angle of resolution (logMAR) unit step of 0.1. To test visual acuity, a single row of four optotypes of the same size was presented simultaneously.

To minimize the total testing time, the potential visual acuity threshold was determined as follows. Visual acuity testing started at the 20/200 line. Only the first optotype in each line was used for testing. After a correct answer, the test administrator proceeded to the next line (e.g., if the answer for the first optotype in the 20/125 line was correct, the test administrator displayed the 20/100 line). After an incorrect answer, the test administrator returned to the first optotype two lines higher (e.g., if the answer for the 20/25 line was incorrect, the test administrator returned to the 20/40 line and performed the standard visual acuity test).

**Figure 4:**
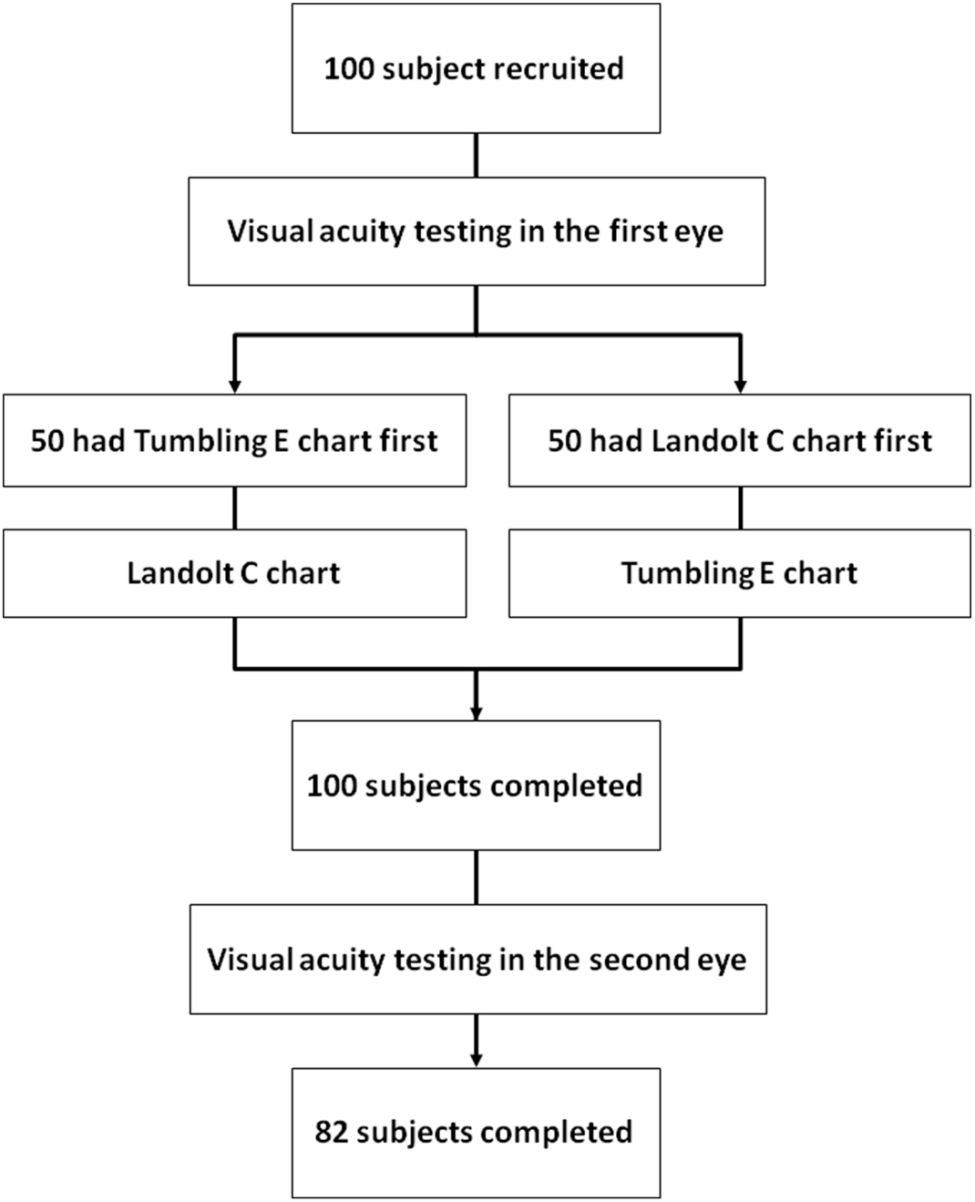
Flowchart of visual acuity testing procedure.

To control for memorization of the visual acuity chart, only the first optotype in each line was used for testing. Regardless of whether the first optotype was correctly identified, another line with same-sized optotypes was shown. If 3 out of 5 same-sized optotypes were correctly identified, the test administrator proceeded to the line with the next smaller optotypes (i.e., the best of 5 for each line). Successively smaller optotypes were used until the child incorrectly identified 3 same-sized optotypes. Vision was recorded as the line above that where the error occurred. The Landolt C and tumbling E charts were used to test one eye at a time (Fig. 4). The order of the visual acuity charts was randomized.

### Significant amblyopia risk factors

Significant amblyopia risk factors were defined as (1) strabismus with strong fixation preference; (2) significant visual axis obstruction or interference (e.g., cataracts, ptosis, etc.); or (3) significant refractive error (anisometropoia > 2.00 D, astigmatism ≥ 1.50 D, hyperopia > 3.50 D, or myopia < − 5.00 D). Refractive errors were identified by cycloplegic retinoscopy.

### Amblyopia criteria

The diagnostic criteria for amblyopia were decreased visual acuity (described below) with the best spectacle correction in place and one of the major amblyopia risk factors mentioned above. The criterion for bilateral amblyopia was Landolt C chart visual acuity (logMAR format) > 0.30 (0.5 in decimal format) at age 3 years, > 0.22 (0.6 in decimal format) at age 4 years, > 0.15 (0.7 in decimal format) at age 5 years, > 0.10 (0.8 in decimal format) at age 6 years or > 0.00 (1.0 in decimal format) at age 7 years. The criterion for unilateral amblyopia was an interocular visual acuity difference of two or more lines. If amblyopia could not be conclusively excluded in a new patient, the amblyopia test was repeated 1–2 times at 2-month intervals.

### Statistical analysis

Visual acuity was transformed to logMAR format for further calculations. Visual acuity scores were compared between the Landolt C and tumbling E charts. Data were compared by paired t test, Wilcoxon signed rank test, t test, or Mann–Whitney U test, depending on the characteristics of the data. The feasibility of testing visual acuity in one eye at a time was also evaluated by McNemar test. For each eye, this study investigated whether amblyopia increased the difference in visual acuity scores between the two charts. Another objective was to determine whether a systemic disease decreased visual acuity scores. The association between age and visual acuity was also investigated. We used Bland–Altman analysis to assess the agreement between the two visual acuity charts. Arbitrary criteria used in analyses of sensitivity and specificity for different years of age included (A) visual acuity (logMAR format): > 0.40 (0.4 in decimal format) at age 3, > 0.30 (0.5 in decimal format) at age 4, > 0.22 (0.6 in decimal format) at age 5, > 0.15 (0.7 in decimal format) at age 6, > 0.10 (0.8 in decimal format) at age 7, or > 0.00 (1.0 in decimal format) at age 8 and older; (B) visual acuity: > 0.30 at age 3, > 0.22 at age 4, > 0.15 at age 5, > 0.10 at age 6 or > 0.00 at age 7 and older; (C) visual acuity: > 0.22 at age 3, > 0.15 at age 4, > 0.10 at age 5, or > 0.00 at age 6 and older; (D) visual acuity: > 0.15 at age 3, > 0.10 at age 4, > 0.05 (0.9 in decimal format) at age 5, > 0.00 at age 6 and older. In ROC curve analysis, the best cutoffs for using the Landolt C chart and tumbling E chart to measure visual acuity were calculated for ages < 84 months and ≥ 84 months. SPSS v14.0 (SPSS Inc, Chicago, IL, USA) was used for all statistical analyses. The hypothesis tests were 2-sided and a p value < 0.05 was considered statistically significant.

## Data Availability

The data included in this study are available from the corresponding author upon reasonable request and after approval by the Institutional Review Board, Kaohsiung Medical University Chung-Ho Memorial Hospital.
